# A Study on Mechanical Characteristics of Phosphor Film Containing Methyl Silicone Resin Based on Crosslinking Reaction Analysis

**DOI:** 10.3390/polym10040370

**Published:** 2018-03-26

**Authors:** Jeong Yeon Park, Young Bae Ko, Moonwoo La, Gil Sang Yoon

**Affiliations:** Molds & Dies Technology R & D Group, Korea Institute of Industrial Technology (KITECH), Incheon 21999, Korea; parkjy@kitech.re.kr (J.Y.P.); kaiser74@kitech.re.kr (Y.B.K.)

**Keywords:** phosphor film, methyl silicone resin, crosslinking reaction, mechanical properties, white LED

## Abstract

This study reveals a methodological research for predicting mechanical properties of phosphor films through the chemical crosslinking reaction of methyl silicone resin during fabrication of the phosphor films. Crosslinking point according to the type of methyl silicone resins was verified through the magnitude of the absorption peak of the functional group and the curing reaction heat. Then, we measured mechanical properties of the fabricated phosphor films. As a result, it was figured out that the number of the crosslinking point was directly proportional to the total curing reaction heat, and also affected the mechanical properties of the phosphor films. Based on the correlation of curing reaction heat and crosslinking point of the methyl silicone resins and mechanical properties of the fabricated phosphor films, we proposed a methodology that can understand and control the phosphor films in advance of finishing the fabrication of the final phosphor products.

## 1. Introduction

Phosphors are materials that absorb light emitted from a light source such as Light Emitting Diodes (LEDs) and convert it to light rays with different wavelengths. Phosphors combined with LED chips use photoluminescence as the energy source to excite the electrons into a state of high energy. In the case of yellow phosphors that are commonly used, it can be classified into oxide phosphors (YAG: Ce) where Ce^3+^ ions are doped with activators and Eu^2+^ ion doped silicate phosphors. Silicate phosphors have a narrow luminescence spectrum compared to oxide phosphors, so their color rendering property is lower. However, silicate phosphors have excellent chromaticity according to the operating current, and thus has the advantage that various wavelengths ranging from green and yellow to orange can be carried out by changing the chemical composition [1,2,3,4,5]. Phosphors are generally used in the form of a film, i.e., a phosphor film, combined with transparent materials such as PMMA (poly methyl methacrylate), epoxy resins, and glass. When phosphor particles are mixed in transparent resins, the ensuing product has enhanced mechanical strength such as hardness and tensile strength, while the tackiness reduces [6,7,8]. Under the tensile load, particle reinforced resin shows strain amplification, which means average local strain exceeding macroscopic strain, because of the mixed particles dissipating the strain energy. Therefore, particle reinforced resin represents higher ultimate strain and stress values than the resin by itself. In addition, the strain amplification increases the average strain rate of resin and has an impact on increasing ultimate stress. As shown in Equation (1), when particle volume fraction inside the resin increases, the strain amplification also increases [9]

(1) $$E=\;E_{0}\left(1+2.5\phi +14.1\phi^{2}\right),\;\epsilon ,\;\dot{\epsilon }\;\to 0,$$

where E represents elastic modulus of resin filled with rigid spherical particles, $E_{0}$ represents elastic modulus of resin without particles, ϕ represents the particle volume fraction, ϵ represents tensile modulus at infinitesimal strain, and $\dot{\epsilon }$ represents the small strain rate. As for particle reinforced resin at a molecular level, the equilibrium retractive force (or contractile force) of resin is proportional to the crosslinking density. As a result of a chemical reaction with help of proper catalysts and cross-linkers, a large number of crosslinking forms simultaneously. This is due to the attachment of several polymer chains on the surface of particles. The increased crosslinking density, in turn, leads to an enhancement of the equilibrium retractive force. Consequently, the tensile strength of the particle reinforced resin becomes much higher than that of the resin by itself [6].

Phosphor converted LED (pc-LED) is a product that combines a LED light source (namely, LED chip) with a phosphor film [10,11,12,13]. Optical properties of the pc-LED, such as luminance, color rendering, and correlated color temperature, depend on the phosphor’s light convergence efficiency. pc-LEDs have been applied in a wide range of fields including cell phones, laptops, tablets, other display units, automotive parts, projectors, and lighting devices [14]. Furthermore, white LED is being used widely in next-generation lighting systems on account of its low power consumption, long life, high luminance, fast response, and eco-friendly properties compared to incandescent lamps [15,16]. The fabrication method of lighting white LED is mainly divided into two categories: the method of combining LED chips with different colors, and the method of combining phosphors in LED chips. In the latter approach, it is categorized into the type of combining red, green and blue phosphors in UV LED chips, and the type of using yellow phosphors in blue LED chips. The method of combining blue LED chips with yellow phosphors offers white LED with excellent luminous efficiency through the simple fabrication and is therefore used in various lighting devices [17,18,19,20].

The pc-LED is generally made by coating a mixture of a phosphor and an uncured transparent resin, namely phosphor slurry, on the LED chip. While a transparent resin with low specific gravity (1.05–1.10 g/cm^3^) is being solidified, phosphors with high specific gravity (4.5 g/cm^3^ or higher) precipitate. The non-uniformly distributed phosphors often lead to defects such as non-uniform color coordination of the fabricated pc-LED [21]. Recently, several methods such as electrophoresis or electrophoretic deposition, adhesion of ceramic phosphor plate, photolithography, coating & volatilization, and self-adaptive phosphor coating have been reported to fabricate phosphor films with even phosphor distribution and to construct systems to mount it on top of the LED [22,23,24,25]. However, these methods show certain drawbacks such as the following: (1) permeation of unexpected materials such as electrolytes and volatile organic compounds during the phosphor film production process; (2) poor adhesive behavior due to differences in the mechanical properties between the phosphor film and the adhesive; and (3) restrictive production scale under wafer level fabrication. To overcome these drawbacks, we proposed a molding & transferring method, comprised of preparing a metallic or elastic mold with cavity, applying and curing the phosphor slurry inside the cavity, transferring the cured phosphor film, and mounting the film on top of the LED chip [26]. Based on the proposed molding technique, fine phosphor film with consistent thickness was produced. We could also fabricate phosphor films with various shapes corresponding to the configuration of the LED chip. In addition, we were able to avoid the permeation of unexpected materials and solve poor adhesion by introducing phosphor film type and adhesive having similar coefficients of thermal expansion. However, other problems, such as ejecting failure of the phosphor film from the mold cavity occurred. The method also encountered handling failure during the transportation, because of the following: providing inappropriate conditions for ejecting, transferring, and mounting the phosphor film based on insufficient optimization of the curing conditions (curing temperatures, reaction heat, etc.) of the phosphor slurry considering the type of the transparent resin; and inaccurate prediction of the mechanical properties of the cured phosphor film. In order to address these problems at a fundamental level, technologies to predict the mechanical properties of the final phosphor film at the very initial production stage are needed. One can expect minimization of defects in the production process by applying the optimal conditions for the major parameters, such as operation load of the eject pin and pick up head and max vacuum adhesiveness of the pick-up head, for ejecting, transferring, and mounting the phosphor film.

In this study, methodological research was used for predicting the mechanical properties of phosphor films through the curing reaction heat of the phosphor slurry. First, the primary factors affecting the magnitude of the curing reaction heat of the phosphor slurry were investigated. We assessed the chemical crosslinking reaction mechanism of three types of methyl silicone resins and the absorption peak of their functional group. Then, the relationship of the curing reaction heat of each phosphor slurry to the mechanical properties (tensile strength, compression breaking strength, adhesion force) of the phosphor film was obtained. Finally, we derived empirical equations to estimate the mechanical strength of the phosphor film based on the curing reaction heat of the resin.

## 2. Materials and Methods

### 2.1. Materials

A silicone resin was used as a transparent material for mixing with phosphors to make a phosphor film. Since silicone resin has the siloxane bond (Si–O–Si) as the main chain, it is coupled with a higher bond energy (106.0 kcal/mol) than the bond energy needed for the C–C bond (86.0 kcal/mol). On account of this higher bond energy, it has better weather resistance and heat resistance than epoxy resin, which is another heat cured resin, and also has lower yellowing and superior electric insulation and chemical stability. Furthermore, in the field of visible lights, the silicone resin has very high light transmittance, so it is receiving attention as a highly transparent material. Unlike the thermoplastic resin, it does not have re-melting, and unlike phosphor films produced through silica sintering, it has similar properties as the adhesive and thus does not lead to phosphor film separation due to the difference of heat expansion coefficients [27]. Silicone resins are classified as methyl silicone, phenyl silicone, vinyl silicone and trifluoropropyl silicone depending on the type of the functional group coupled with the main chain. Methyl silicone resins, used in this study, have almost no change in light transmittance even when the material is exposed for long periods to environments of 180 °C compared to other resins, and thus guarantees the long-term reliability of the LED package [28,29,30,31].

In order to compare the curing properties and mechanical properties of phosphor films, this study prepared three types of methyl silicone resins (IV70, IVS4752, XE14-C2032; Momentive, Waterford, NY, USA) with a similar viscosity at 4000 cP as shown in Table 1 Each silicone resin was made by mixing a liquid component (liquid A) containing base resin and Pt catalyst (polydimethylsiloxane containing the vinyl group and the Pt(-complex) catalyst) and another liquid component (liquid B) containing base resin and crosslinker (polydimethylsiloxane terminated with the vinyl group and the Si–H oligomer crosslinker) at a weight ratio of 1:1. Then the mixture was cured by applying heat. While curing, the double bond of the vinyl group in the base resin was broken due to the Pt(-complex) catalyst and it formed bonds with the Si–H of the cross-linker, resulting in crosslinking. The curing procedure of such methyl silicone resin made it possible to observe and measure the curing reaction heat generated by the exothermic reaction [1,27]. The properties of the three types of methyl silicone are as shown in Table 1 The mixing viscosity was measured at the fixed spindle shear rate (2.5 s^−1^) under a thermostatic bath (23 °C) connected with a rotational viscometer (DV-I prime, Brookfield, WI, USA) based on the KS M ISO 3219 standard. For shore hardness, the measured value of the manufacturer was taken as reference. The average molecular weight was determined by gel permeation chromatogram (GPC) results using a silicone resin dissolved in tetrahydrofuran (THF) compared with the reference material (polystyrene).

**Figure 1:**
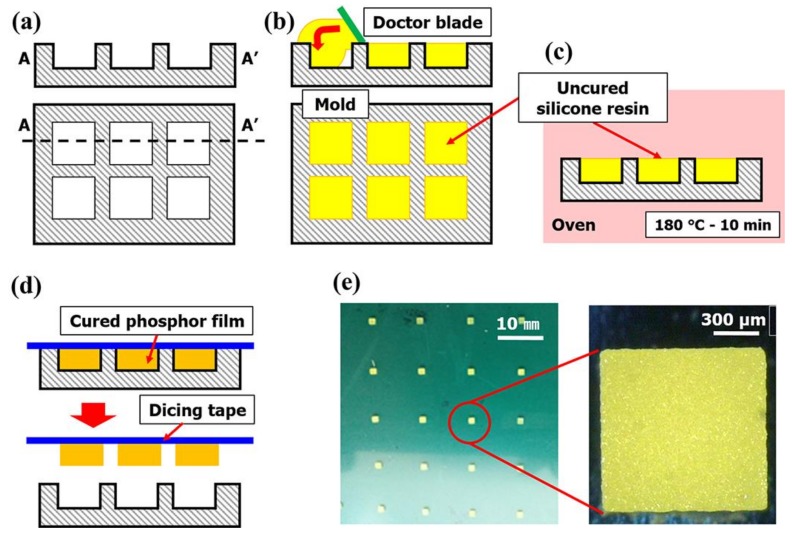
Overview of the phosphor film fabrication process (**a**) design and fabrication of elastic mold; (**b**) phosphor slurry dosing; (**c**) thermal curing; (**d**) phosphor film transferred to dicing tape; (**e**) fabricated phosphor film.

The phosphor slurry was prepared by injecting 30 wt % silicate-based yellow phosphor (EY4453, ρ = 4.7 g/cm^3^, D50V, 15.5 μm, Intematix Co., Fremont, CA, USA) into each resin. The particles were homogeneously distributed in the phosphor slurry using a vacuum mixer. The first mixing condition was the revolution of 1200 rpm, rotation of 500 rpm, and mixing time of 1 min; and the second mixing condition was the revolution of 800 rpm, rotation of 500 rpm, the vacuum pressure of 2.5 bar, and mixing time of 3 min. The detailed process for fabricating the phosphor film is as shown in Figure 1. A silicone rubber (SH0170U, KCC, Seoul, Korea) was used to make an elastic mold with a 1.0 mm × 1.0 mm × 0.3 mm engraved rectangle (Figure 1a). Then, the phosphor slurry was applied by the doctor-blading method in the rectangular cavities of the elastic mold and thermally cured (Figure 1b,c). The cured phosphor slurry with rectangular shape, namely a phosphor film, was transferred from the mold to the dicing tape (base: PVC, adhesive: acrylic, thickness: 0.08 mm, adhesion force: 1.5 N per 20 mm) and used for the determinations of curing reaction heat and mechanical properties (Figure 1d).

### 2.2. Measurements for Chemical Properties

The mechanical properties of the phosphor film were assumed to be closely related to the curing reaction heat. That is, as the crosslinking reaction of the silicone resin proceeds much more, the curing reaction heat increases, and thus the mechanical properties of the phosphor film could be improved. In order to identify the reasons why the curing reaction heat characteristics differ among the various silicone resins, the crosslinking absorption peaks (Si–H) of the silicone resins were compared using Fourier transform infrared spectrometer (FT-IR). Each resin was coated on a glass and cured at 180 °C for 10 min to form a film type specimen. Then, the absorption peaks of the wavelength band of each resin were observed in the FT-IR of ATR. Thermal analysis by the differential scanning calorimetry (DSC) was performed to derive the total curing reaction heat of each phosphor slurry according to the resin type. The analysis conditions, based on KS M ISO 11375 specifications, applied a heating rate of 5, 10, 15, or 20 °C per min from 30 to 200 °C, under a N_2_ atmosphere. The cure onset temperature (or onset temperature of curing), peak cure temperature, end cure temperature and the total curing reaction heat of each phosphor slurry were investigated by measuring the heat flow according to the temperature. The activation energy was derived from the isothermal reaction speed equation, Equation (2), or the heat-rising reaction equation, Equation (3), and the curing reaction kinetics related to the autocatalysis [32,33]

(2) $$\frac{d\alpha }{dt}=k\alpha^{m}{\left(1-\alpha \right)}^{n},\;k=A\exp\left(-\frac{E_{a}}{RT}\right),$$

(3) $$\frac{d\alpha }{dT}=\frac{1}{\beta }\alpha^{m}{\left(1-\alpha \right)}^{n},$$

where dα/d*t*, α, *k*, *m*, *n*, *A*, *R*, *E_a_*, *T*, and β indicate the degree of curing changes with time, the degree of curing, the curing reaction constant, the 1st reaction order, the 2nd reaction order, the frequency factor, the gas constant, the activation energy, the absolute temperature, the heating rate(d*T*/d*t*), respectively. Constants *k*, *m*, *n* can be found through non-linear regression analysis. Frequency constant (*A*) and activation energy (*E_a_*) determined from the Kissinger’s equation as below

(4) $$-\ln\left(\frac{\beta }{T_{p}{}^{2}}\right)=\frac{E_{a}}{RT}-\ln\left(\frac{AR}{E_{a}}\right),$$

where $T_{p}^{2}$ is the peak cure temperature.

The curing conditions of 12 cases as shown in Table 2 were set in order to derive the optimal curing condition when the phosphor film was formed using the phosphor slurry of which the total heat of curing reaction was confirmed. Here, $Q_{T}$ refers to the total curing reaction heat or the reaction heat of phosphor slurry in uncured state as measured in the previous paragraph, and $Q_{i}$ represents the remaining curing reaction heat measured when applying the curing process conditions of Table 2. The degree of curing of the phosphor film can be calculated by the total heat of curing reaction of the phosphor slurry and the remaining curing reaction heat after the phosphor film forming ($\alpha =\left(Q_{T}-Q_{i}\right)/Q_{T}$).

**Figure 2:**
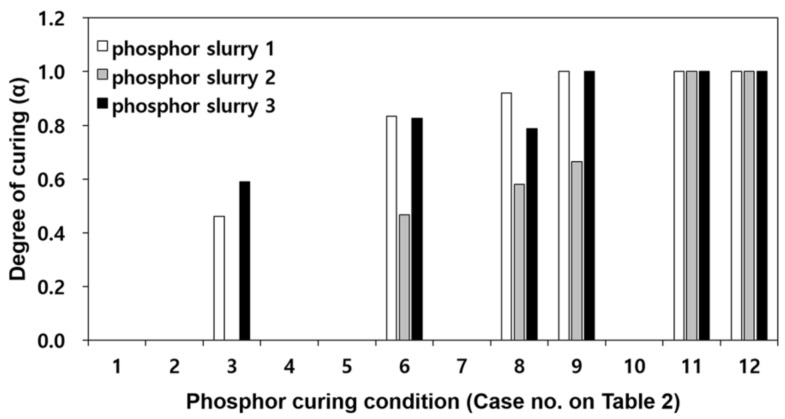
Comparison of the degree of curing of phosphor film per phosphor slurry and curing process conditions.

Figure 2 is a comparison of the degree of curing for phosphor films when applying the curing process conditions of Table 2 for the three types of phosphor slurries. For cases 1, 2, 4, 5 and 7, not enough curing occurred that could cause ejection of the phosphor film, and therefore, the remaining curing reaction heat could not be measured. Regardless of the phosphor slurry type, the minimum degree of curing where the phosphor film could eject from the elastic mold was found to be *α* = 0.46. In all three phosphor slurries, when heated for more than 5 min (case 12) at curing temperature of 180 °C, the phosphor films were completely cured. Therefore, in order to measure the mechanical properties of phosphor films molded per resin, the specimen molding condition was set at curing temperature of 180 °C and curing time of 10 min.

### 2.3. Measurements for Mechanical Properties

During the phosphor film ejection/transfer process, the crucial mechanical properties are the tensile strength, compression breaking strength, and adhesion force with the mold. The tensile strength is closely related to the product features of the phosphor film and the phosphor film ejection and pick up equipment used to mount the film from the dicing tape or elastic mold to the top of the LED chip. During phosphor film ejection from the dicing tape or elastic mold, the vacuum adhesion pad located below the phosphor film pulls on the bottom of the phosphor film, and if tensile strength of the phosphor film is excessively low, bending (or warpage) can occur during ejection or transfer, making it difficult to mount the film on the desired location. In addition, even after adhesion with the LED luminescence part, when the LED chip reaches high temperatures, bending may occur due to the remaining stress accumulated during the ejection and transfer process. This may cause the gap between the LED luminescence part and the phosphor film to become wider, reducing the ratio of conversion to yellow light. As a result, the white that was set as the goal during the design of the LED element will not be realized. On the other hand, if the tensile strength of the material making up the phosphor film is known in advance, it is possible to determine the max vacuum adsorption strength of the vacuum adhesion pad during ejection and transfer of the phosphor film, and thereby compute the optimal operating load of the eject pin and pick up head. This way, the phosphor film can be mounted precisely on the LED chip without damaging or bending. In addition, during phosphor film ejection from the dicing tape or elastic mold in the phosphor film ejection/transfer process, the pick-up head on top of the phosphor film comes into contact with the upper part of the phosphor film and then raises the phosphor film through vacuum adhesion. At this time, if the compression breaking strength of the phosphor film is excessively high, then brittle fracture in compression can occur due to the pick-up head, and if the compression breaking strength of the phosphor film is excessively low, it may cause the brittle fracture in tension due to the pick-up head. Thus, the compression breaking strength of the phosphor film also has a close relationship to the phosphor film ejection/transfer process features. Lastly, the adhesion force between the phosphor film and the mold for fabricating phosphor films has a close relationship with the eject/transfer process features of the phosphor film. If the adhesion power between the phosphor film and mold is too high when transferring or ejecting the phosphor film from a metal mold or elastic mold cavity with a dicing tape, the phosphor film will not be separated from the mold. Therefore, we investigated the tensile strength, compression breaking strength, and adhesion force of the fabricated phosphor film, in order to identify the correlation between the total curing reaction heat and mechanical strength when the phosphor film produced is completely cured (*α* = 1). In this investigation for the mechanical strength, we designated the phosphor films fabricated from resin 1, 2 and 3 as specimens 1, 2, and 3, respectively.

**Figure 3:**
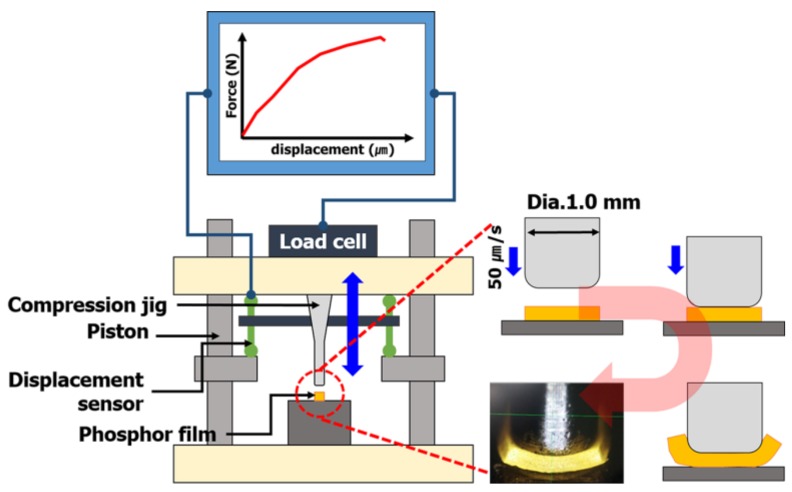
Compression breaking strength measurement diagram.

**Figure 4:**
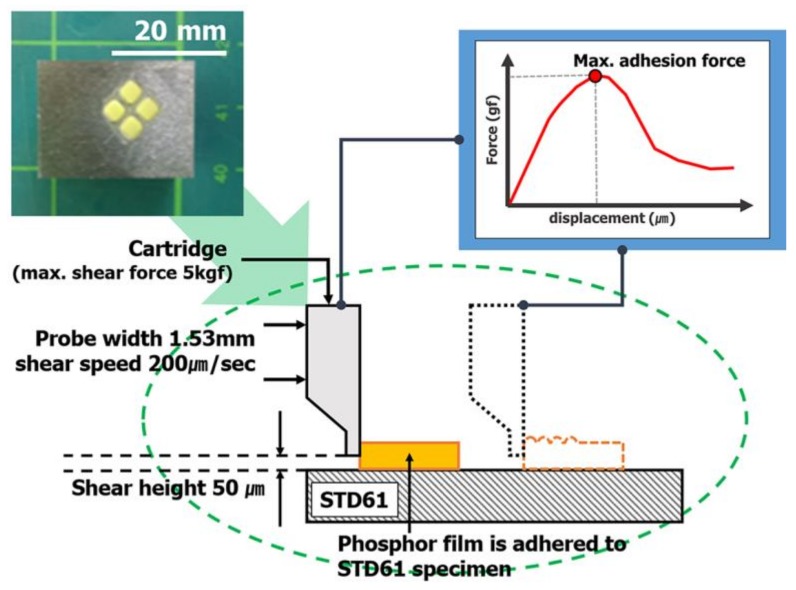
Principle of shear testing for measuring the adhesion force between the phosphor film and STD61.

Specimens for measuring tensile strength to predict the tensile strength of phosphor films using three types of phosphor slurries were fabricated. The specimens for measuring tensile strength, based on the specifications of ASTM D412, injected 5.6 g of 3 phosphor slurries in which the tensile specimen shape was engraved in a metal mold (STD61, PTFE coated). Using a tensile tester (KNR Systems, Yongin, Korea), each specimen was pulled at 23 °C and a tensile speed of 50 mm/min to compare the stress results according to the deformed length. In particular, the tensile strain and ultimate tensile stress up to the point of breaking in the stress–strain curve for each of the three resins were compared with the tensile strength (tensile strength at 5% elongation) when pulled by 5%. The compressive fracture strength of the phosphor film was measured using a micro-compression device (KNR Systems, Yongin, Korea) with a metal jig having a diameter of 1 mm. The compressive load (N) per jig transfer distance was measured when the top surface of the phosphor film was pressed with 0.05 mm/s speed to a breaking point of 0.3 mm (Figure 3). The solder ball shear test (JESD22-B117) was introduced to investigate the adhesion power between the elastic mold and the cured phosphor film. Phosphor slurry was applied at a size of 2.0 mm × 2.0 mm × 0.3 mm on top of a metal specimen (STD61) as shown in Figure 4 and then cured by applying the curing conditions determined in Section 2.2. After applying a shear load (speed of 200 μm/s and height of 50 μm) at the side of the phosphor film using the bonding tester (Dage 4000, Nordson Dage, Buckinghamshire, UK), the maximum shear load immediately before peeling was measured to verify its adhesion power.

## 3. Results and Discussions

### 3.1. Functional Group Absorption Peak Analysis

**Figure 5:**
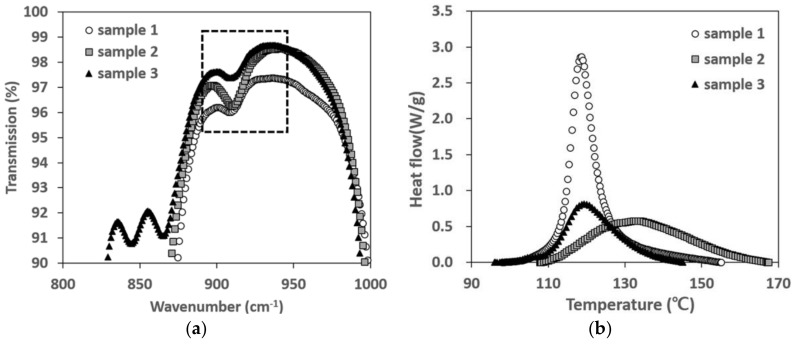
(**a**) Comparison of absorption peaks of the three resins: to be drawn by narrowing to 800–1000 cm^−1^ and (**b**) DSC heat flow result among the 3 resins.

Figure 5a shows 1000–800 cm^−1^ among the absorption peaks of the three resins according to the infrared wavelength in FT-IR. Of the absorption peaks shown in Figure 5a, the part that indicated 950–880 cm^−1^ in the dotted box for Si–H pertains to the crosslinker where the crosslinking reaction displayed the absorption peak [34,35]. Within the indicated range, resin 1 has the lowest transmission compared to the other two resins. It means that the absorption peak of the resin 1 for the corresponding wavelength is the largest. As a result, it can be seen that the number of crosslinking reactions is relatively large in the order of resin 1, 2, and 3. For reference, the relationship of the absorption peak and functional group per wavelength are shown in Figure S1 and Table S1 (Supplementary Materials).

### 3.2. Curing Reaction Heat of Phosphor Slurries

Figure 5b is a graph that shows the heat flow (W/g) per unit mass according to temperature changes for the three types of silicone resins (resin 1–3). As mentioned earlier, the curing reaction of methyl silicone resins are exothermic so the heat flow value is a positive number. By calculating the width of the exothermic graph from the cure onset temperature to the end cure temperature per unit mass, it is possible to calculate the total curing reaction heat for the corresponding resin. Upon calculations, the total curing reaction heat for resin 1 was obtained for 27.31 J/g, the total curing reaction heat of resin 2 was 18.54 J/g, and the total curing reaction heat of resin 3 was 14.57 J/g (also see Figures S2–S4, Supplementary Materials); thus, the total curing reaction heat of resin 1 was up to 1.8 times higher than that of the other two resins. As mentioned in Section 3.1 and Figure 5a, resins in which Si–H curing reactions were observed the most were in the order of resin 1 > resin 2 > resin 3, and the total curing reaction heat was also in the order of resin 1 > resin 2 > resin 3. Therefore, the factor that has the greatest influence on the curing reaction is the frequency of Si–H within the silicone resin, not the average molecular weight of the silicone resin. The curing reaction constant value of each resin are as shown in Table 3 The magnitude of the activation energy of the three types of silicone resins was in the order of resin 1 > resin 2 > resin 3. This means that when the frequency of Si–H is high in the silicone resin, activation energy needed for curing reactions becomes larger.

### 3.3. Tensile Strength of Phosphor Films

**Figure 6:**
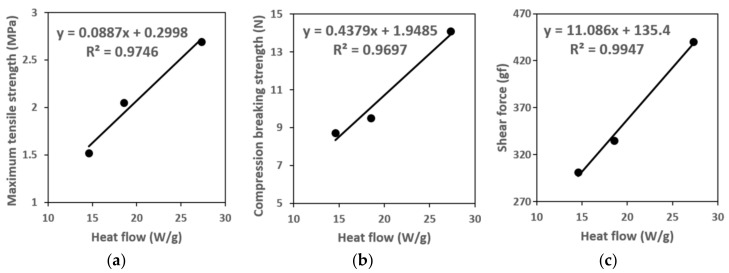
Results of mechanical tests: (**a**) relationship between curing reaction heat and ultimate tensile strength; (**b**) comparison of total heat of cure reaction and compression breaking strength of phosphor film per resin; and (**c**) comparison of total heat of cure reaction and shear force of phosphor film per resin.

In the case of tensile strain (%) up until the rupturing point for the three types of phosphor slurries, it increases as the average molecular weight of the silicone becomes higher. It was confirmed that tensile strain was not related to the curing reaction heat or surface strength of the phosphor resin. However, specimen 1, which had the largest total curing reaction heat, was found to also have the largest ultimate tensile strength at 2.69 MPa, and specimen 2 was measured at 2.05 MPa and specimen 3 at 1.52 MPa, and it was thus consistent with the order of the size of the total curing reaction heat. When comparing the value of tensile strength at 5% elongation, which is the index that substitutes Young’s modulus, specimen 2 that had the lowest breaking strength at peak (%) value measured significantly higher at 26.36 MPa, and was followed in the order of specimen 1 at 7.74 MPa and resin 3 at 1.05 MPa. In the case of Young’s modulus, in addition to the frequency of the Si–H functional group, the effect of average molecular weight was not consistent with the order of magnitude of the total curing reaction heat. Furthermore, it was confirmed that the comparison of ultimate tensile strength followed the same pattern as the order of the total heat of cure reaction size as shown in Figure 6a. In other words, the ultimate tensile strength (*y*) of each phosphor slurry was proportionate with the total curing reaction heat (*x*). When expressing this as an equation, we can obtain the relational equation of *y* = 0.0887*x* + 0.2998 (*R*^2^ = 0.9746).

### 3.4. Compression Breaking Strength of Phosphor Films

Figure 6b illustrates the comparison of the total curing reaction heat and compression breaking strength (N) when fabricating the phosphor film using phosphor slurries applied to each resin. For reference, the relationship of the resulting force and compression displacement are shown in Figure S5 (Supplementary Materials). The compression breaking strength and the total curing reaction heat had proportional relations, and it was found that there was no relationship between compression breaking strength and average molecular weight. Furthermore, the functional group (Si–H) frequency order and the total curing reaction heat magnitude order, as well as the compression breaking strength size order showed consistent patterns for specimen 1, specimen 2 and specimen 3. Also, the relational equation of *y* = 0.4379*x* + 1.9485 (*R*^2^ = 0.9697) was established between the total heat of cure reaction (*x*) and compression breaking strength (*y*).

### 3.5. Shear Force of Phosphor Films

Figure 6c compares the total curing reaction heat and shear force (gf) when fabricating the phosphor film by applying each resin. Shear force was also seen to be proportional to the total curing reaction heat, and when considering only the shear force, it is lowest when applied to resin 3 of the three resins, and therefore, it is judged that it would be most advantageous to use resin 3 in the phosphor film eject and transfer processes. There is a relationship of *y* = 11.086*x* + 135.4 (*R*^2^ = 0.9947) between the total curing reaction heat (*x*) and shear force (*y*). Like other mechanical properties, it was confirmed that the functional group (Si–H) frequency order, total heat of cure reaction size order, and shear force size order showed consistent patterns in the order of specimen 1, 2, and 3.

## 4. Conclusions

This study pursued research on methods to predict the mechanical properties of phosphor films through curing reaction heat that appears in the manufacturing of phosphor films. Capabilities to predict and adjust mechanical properties would be useful for technological development that could control the mechanical properties of phosphor films. For this, the correlation between the chemical crosslinking mechanism of three types of methyl silicone resins, size of the absorption peaks of the functional groups, and curing reaction heat of phosphor slurries were analyzed to investigate the factors that affect the magnitude of curing reaction heat. Next, the relationship of curing reaction heat of each phosphor slurry and the mechanical properties (ultimate tensile strength, compression breaking strength, adhesion force) of phosphor films was analyzed, and an empirical equation that could predict the mechanical strength of phosphor films by measuring the heat of curing reaction heat was deduced. It was found that the property of silicone resins that impacts the mechanical properties of phosphor films was not average molecular weight, but the frequency of functional groups (Si–H) within the cross-linker. It was verified that, as the crosslinking points increased when the frequency of the silicone resin’s Si–H functional group grew, the total curing reaction heat in the phosphor slurry that applied this rose. This, in turn, improved the mechanical properties of the phosphor film produced. Using the empirical relational equation between the total curing reaction heat and mechanical properties found in this study, it became possible to predict the mechanical properties; it only required measuring the curing reaction heat via DSC. It was also possible to set the optimal curing temperature needed for fabricating the phosphor film. Furthermore, it is expected that it will be possible to predict whether there will be damage to a phosphor film with a mechanical strength value calculated using the empirical equation designed through this study, if it is mounted on top of the LED chip using a phosphor film ejection and pick up equipment that operates in the range of (eject pin’s maximum operation load range (<200 N) and pick up head’s maximum vacuum adhesion force (<1 gf). Therefore, additional research is scheduled in the future for quantifying the correlation between the phosphor films’ ejection/transfer process parameters and phosphor film product features. Ultimately, it is expected that by raising tensile strength/compression breaking strength and conducting research on the optimal mixing ratio of different silicone resins to fabricate phosphor films with low adhesion power, it will be possible to develop technologies that can control the mechanical properties of phosphor films.

## Figures and Tables

**Table 1:** Three types of methyl silicone resins.

Property	Resin 1	Resin 2	Resin 3
Mixing viscosity (cP)	4537	4033	4941
Shore hardness	A70	A70	A43
M_n_^1^, liquid A	2130	2130	2090
M_n_, liquid B	1800	1450	1710
M_w_^2^, liquid A	22,700	8320	24,900
M_w_, liquid A	27,800	5230	39,500
M_w_/M_n_, liquid A	10.66	3.9	11.92
M_w_/M_n_, liquid A	15.44	9.6	23.12

^1^ Molecular number, ^2^ Molecular weight.

**Table 2:** Experiment plans to observe curing per curing process conditions of phosphor films.

Case No.	1	2	3	4	5	6	7	8	9	10	11	12
Temperature (°C)	100	100	100	120	120	120	150	150	150	180	180	180
Time (min)	1	5	10	1	5	10	1	5	10	1	5	10

**Table 3:** Constants of curing reaction kinetics described in Equations (2)–(4).

	Resin 1	Resin 2	Resin 3
*k*	0.1293	0.01067	0.03222
log *A*	13.84	10.93	9.655
*E_a_* (J/g)	114.92	97.70	84.57
*m*	1.329	0.5771	0.9345
*n*	2.01	0.7202	10.09
*R*^2^	0.9917	0.9987	0.9778

## Supplementary Materials

The following are available online at http://www.mdpi.com/2073-4360/10/4/370/s1, Figure S1: Comparison of absorption peaks of the three resins, Figure S2: Comparison of heat flow vs. temperature per heating rate for calculating the activation energy of Resin 1, Figure S3: Comparison of heat flow vs. temperature per heating rate for calculating the activation energy of Resin 2, Figure S4: Comparison of heat flow vs. temperature per heating rate for calculating the activation energy of Resin 3, Figure S5: Resulting force vs. compression displacement for Resin 1, 2, and 3, Table S1: Absorption peak of each resin and functional group in absorbance areas.
