# Supplementary material for: A Study on Mechanical Characteristics of Phosphor Film Containing Methyl Silicone Resin Based on Crosslinking Reaction Analysis
Source: Polymers (Basel). 2018 Mar 26;10(4):370. doi: 10.3390/polym10040370 (PMC6415198; doi:10.3390/polym10040370)
Supplement: Supplementary file 1 [file polymers-10-00370-s001.pdf]

Supplementary Materials

A Study on Mechanical Characteristics of Phosphor Film Containing Methyl Silicone Resin based on Crosslinking Reaction Analysis

Jeong Yeon Park [1], Young Bae Ko [1], Moonwoo La [1,*], and Gil Sang Yoon [1,*]

[1] 1Molds & Dies Technology R&D Group, Korea Institute of Industrial Technology (KITECH), Incheon 21999, Korea; parkjy@kitech.re.kr (J.Y.P.); kaiser74@kitech.re.kr (Y.B.K.); mla@kitech.re.kr (M.L.); seviaygs@kitech.re.kr (G.S.Y.)

* Correspondence: mla@kitech.re.kr; +82-32-8500-340 and seviaygs@kitech.re.kr; +82-32-8500-335

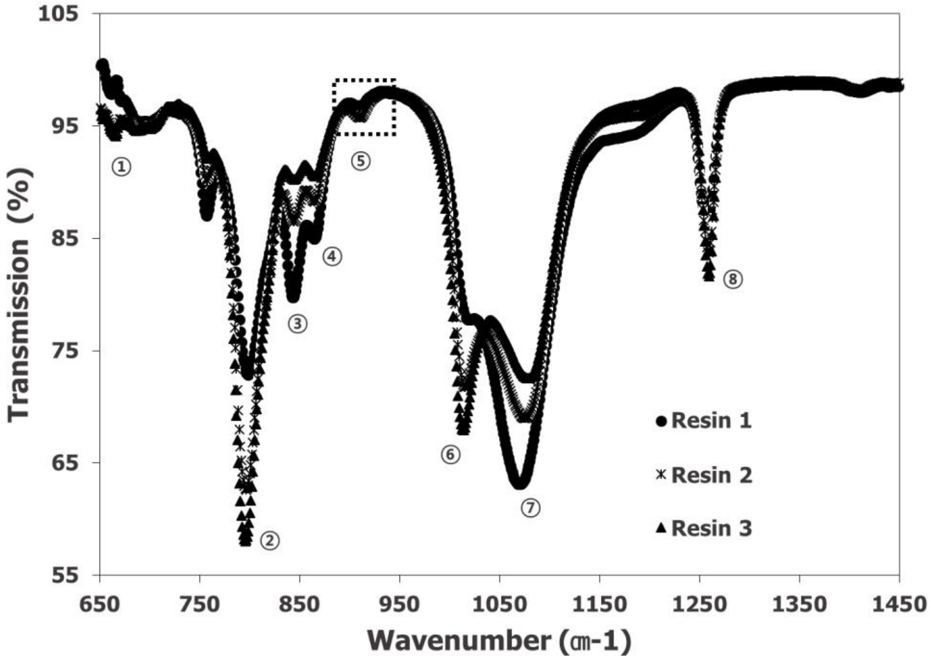

Figure. S1. Comparison of absorption peaks of the three resins.

Table S1. Absorption peak of each resin and functional group in absorbance areas

Absorbance Area	Resin 1	Resin 2	Resin 3	Functional group
①	757.423	756.836	Indefinite	CH non-coplanar bending
②	796.486	798.052	795.814	>Si(-CH_3)$_2$
③	844.165	843.249	844.328	Si-O stretching
④	864.523	864.214	865.087	Si-O stretching
⑤	909.977	908.032	908.85	Si-H deformation and wagging
⑥	1015.14	Indefined	1013.84	SiO_3
⑦	1015.14	1069.92	1078.43	Si-O-Si
⑧	1258.88	1257.53	1259.11	>Si(-CH_3)$_2$

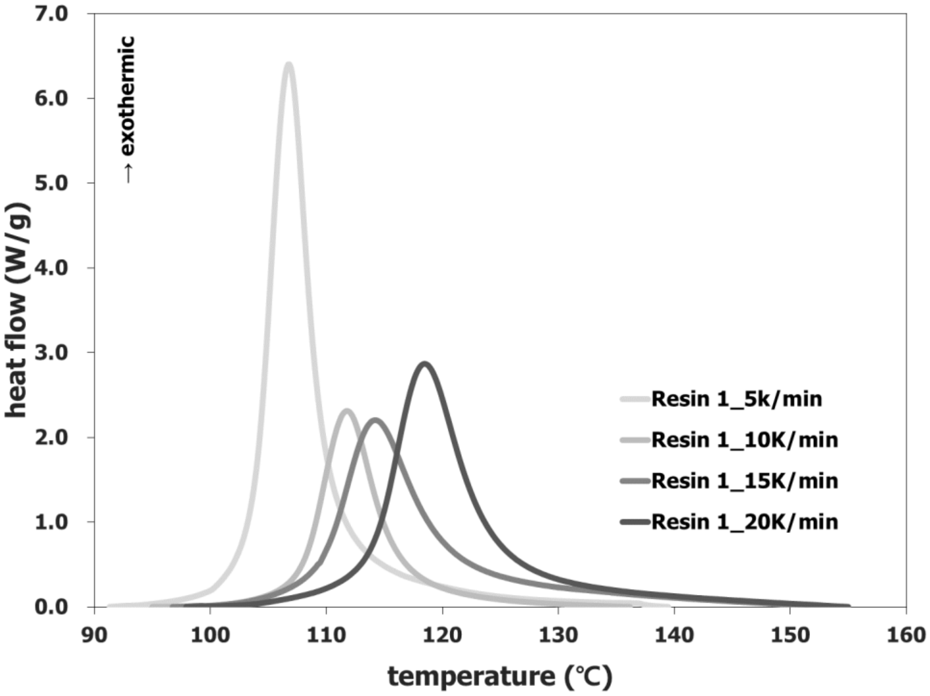

Figure. S2. Comparison of heat flow vs. temperature per heating rate for calculating the activation energy of Resin 1.

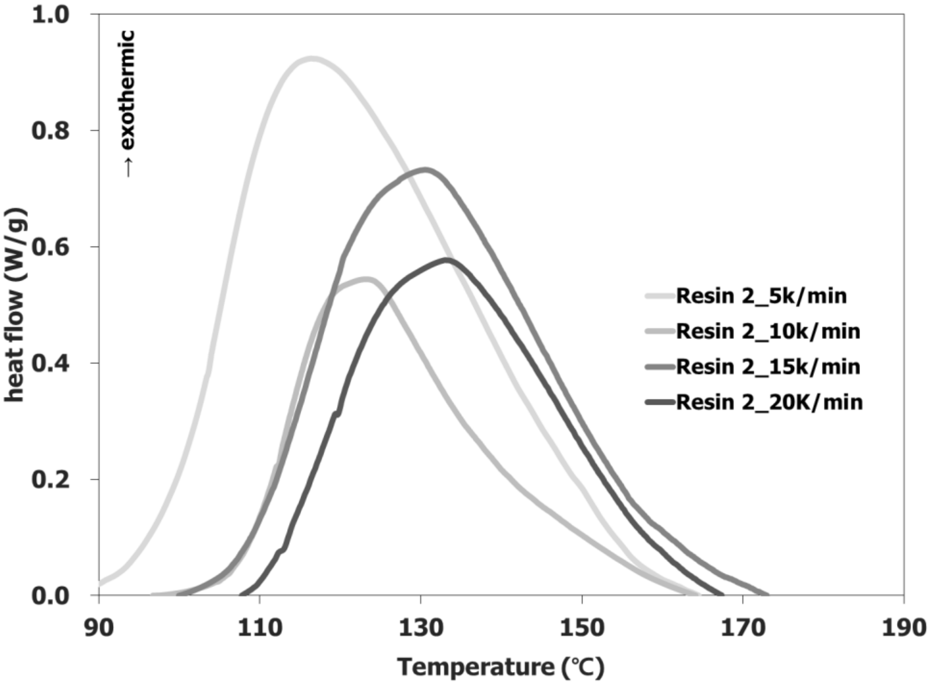

Figure. S3. Comparison of heat flow vs. temperature per heating rate for calculating the activation energy of Resin 2.

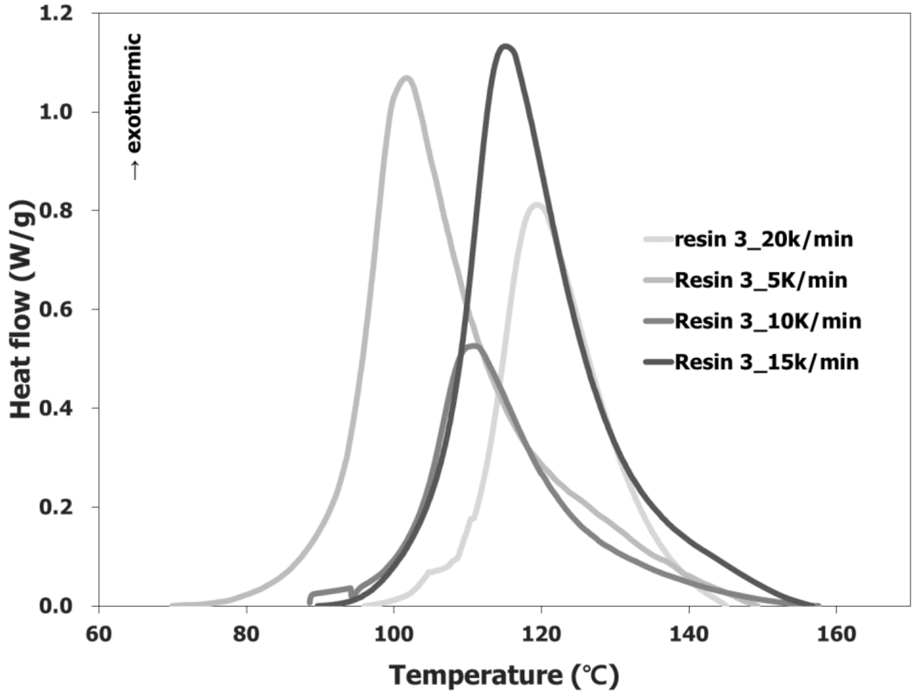

Figure. S4. Comparison of heat flow vs. temperature per heating rate for calculating the activation energy of Resin 3.

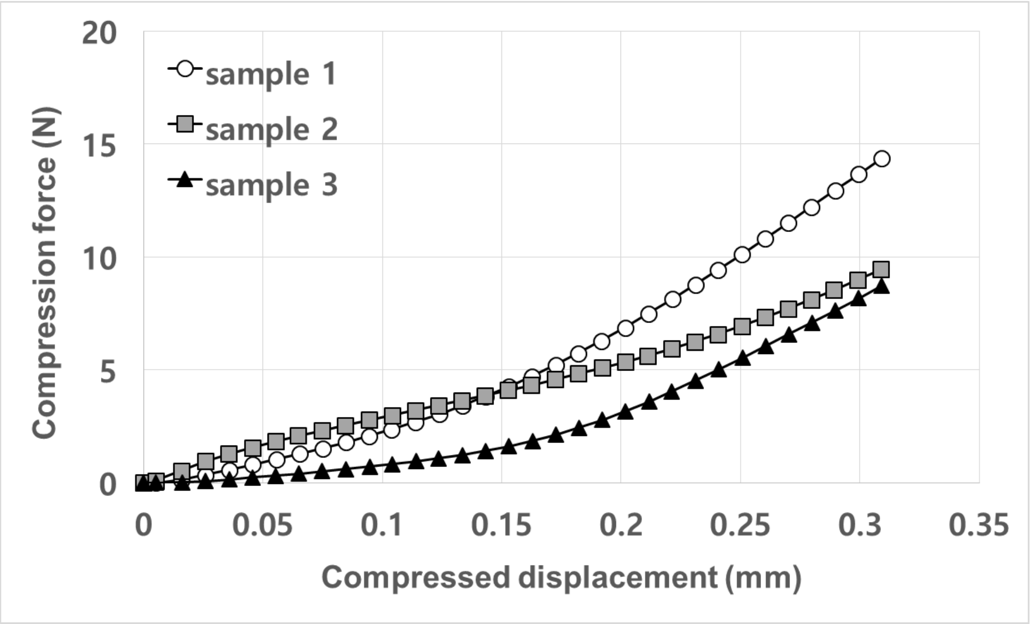

Figure. S5. Resulting force vs. compression displacement for Resin 1, 2, and 3.
